# Pulmonary *Rhizopus arrhizus* infection treated with high-dose liposomal amphotericin B in a heart transplant recipient under ECMO: a case report

**DOI:** 10.3389/fmed.2025.1631873

**Published:** 2025-09-16

**Authors:** Wei Jiang, Jinghui Zhai, Boyu Li, Jie Ma, Sixi Zhang

**Affiliations:** ^1^Department of Clinical Pharmacy, The First Hospital of Jilin University, Changchun, Jilin, China; ^2^Second Clinical Medical College, Harbin Medical University, Harbin, Heilongjiang, China; ^3^School of Pharmaceutical Science, Jilin University, Changchun, Jilin, China

**Keywords:** liposomal amphotericin B, pulmonary *Rhizopus arrhizus*, cardiac transplantation, ECMO support, early diagnosis and treatment

## Abstract

Pulmonary mucormycosis caused by *Rhizopus arrhizus* is an emergent, fulminant threat in immunocompromised hosts, yet therapeutic success remains elusive when extracorporeal membrane oxygenation (ECMO) is required. While liposomal amphotericin B (L-AMB) is endorsed as first-line therapy, its pharmacokinetics are profoundly altered by ECMO—dilution, circuit sequestration, and impaired lung penetration all conspire to sub-therapeutic exposure. We report the first documented case in which these challenges were systematically overcome. A 52-year-old cardiac-transplant recipient, supported on veno-venous ECMO for refractory hypoxaemia, developed rapidly progressive pneumonia. Metagenomic next-generation sequencing (mNGS) of bronchoalveolar lavage fluid returned a definitive *Rhizopus arrhizus* signature within 24 h, prompting immediate escalation to high-dose L-AMB (10 mg/kg/day). Therapeutic drug monitoring confirmed sustained trough levels above 7 μg/mL despite a 3.5-fold increase in volume of distribution. Serial mNGS quantification demonstrated a logarithmic decline in fungal reads to undetectable levels by day 10, accompanied by radiological resolution and preserved renal function. After 28 days of intravenous therapy, the patient was discharged on oral isavuconazole with no relapse at 6 months. This case establishes that early pathogen identification by mNGS, coupled with aggressive L-AMB dose optimisation under rigorous pharmacokinetic guidance, can achieve cure of pulmonary mucormycosis even in the most pharmacologically hostile environment of ECMO support.

## Introduction

Mucormycosis is a life-threatening, invasive fungal disease caused by *Mucorales*, with Rhizopus species in most cases. It almost invariably strikes hosts whose immunity is compromised—diabetes, hematologic malignancies, or solid-organ transplants.

Our patient belongs to the last category. Within weeks of cardiac transplantation, he developed rapidly progression pulmonary *Rhizopus arrhizus* infection (1, 2). Bronchoalveolar lavage fluid was therefore subjected to metagenomic next-generation sequencing (mNGS), which, within 48 h, unequivocally identified *Rhizopus arrhizus* and allowed us to initiate targeted antifungal therapy without delay (3).

The clinical course was complicated further by respiratory failure necessitating veno-venous extracorporeal membrane oxygenation (ECMO). Because ECMO circuits can sequester lipophilic drugs and alter their pharmacokinetics, we escalated to a high-dose liposomal amphotericin B (L-AMB) regimen. Therapeutic drug monitoring confirmed adequate plasma exposure, and serial imaging documented progressive radiological improvement.

Consequently, this case highlights two pivotal lessons. First, rapid pathogen identification via mNGS can compress the diagnostic window and inform precise therapy in immunocompromised hosts. Second, dose optimization of L-AMB remains feasible and effective even under ECMO, providing a practical template for managing similar high-risk scenarios.

## Case presentation

### Chief complaint

Intermittent dyspnea for 2 years.

### History of present illness

The patient was admitted on March 17, 2024, with a diagnosis of cardiomyopathy. Human data were approved by Ethics Committee of the First Hospital of Jilin University (No. 2025-226). Two years prior, he experienced dyspnea and was diagnosed with acute myocardial infarction at another hospital, followed by emergency percutaneous coronary intervention (PCI). Despite this, he continued to have intermittent dyspnea and bilateral lower limb edema. Eighteen months ago, his symptoms worsened, with severe dyspnea, fever, and worsening edema. He was diagnosed with ischemic cardiomyopathy at our hospital and recommended for heart transplantation. He is now admitted for heart transplantation.

### Physical examination

On admission, the patient’s temperature was 36.6 °C, pulse rate was 79/min, respiration rate was 14/min, and blood pressure was 106/71 mmHg. The patient was in a generally acceptable condition. He had undergone coronary stent implantation 1 year ago due to acute myocardial infarction. There was no history of trauma or other past medical history.

### Laboratory examinations

Relevant laboratory examinations were as follows: White blood cell count: 9.53 × 10^9^/L; Neutrophil percentage: 71%; Procalcitonin: <0.05 ng/mL; Creatinine: 80.6 μmol/L.

### Preliminary diagnosis

Ischemic cardiomyopathy; Coronary atherosclerotic heart disease, post-coronary stent implantation; Valvular heart disease; Severe mitral regurgitation; Severe tricuspid regurgitation; Heart failure, NYHA class IV; Pulmonary hypertension; Aortic dissection formation.

### Treatment

On March 18th, the patient underwent cardiac transplantation and started empiric broad-spectrum antimicrobial therapy with cefoperazone-sulbactam and vancomycin. The next day, venoarterial ECMO was implemented for circulatory support. Given the elevated infection markers, persistent fever, leukocytosis, and elevated neutrophil percentage, the antimicrobial therapy was escalated to meropenem. Echocardiography revealed significant cardiac dysfunction: left ventricular wall thickening, segmental wall motion abnormalities, reduced ventricular function, moderate tricuspid regurgitation, and increased pulmonary artery pressure.

On March 21st, the patient’s temperature decreased 37.3 °C, and inflammatory markers showed improvement. However, NGS identified multiple pathogens, including *Klebsiella pneumoniae*, *Rhizopus arrhizus*, *Aspergillus flavus*, *Ureaplasma urealyticum*, and *Ureaplasma parvum*, while blood culture confirmed Carbapenem-Resistant *Acinetobacter baumannii* (CRAB) (Table 1). Vancomycin was discontinued due to the absence of positive cocci. Based on the Infectious Diseases Society of America guidelines, polymyxin (500,000 IU/12 h) and metacycline (loading dose 200 mg daily, maintenance dose 100 mg daily) were administered for CRAB and *Ureaplasma* coverage. For mucormycosis, high-dose L-AMB (10 mg/kg daily) and amphotericin B cholesterol sulfate complex nebulization were initiated, in accordance with European Confederation of Medical Mycology recommendations.

**Table 1:** Drug adjustments and indicator changes during antifungal treatment.

Index/Days	D3	D4	D5	D9	D10	D13	D14	D22	D25	D29	D32	D37	D43	D51	D55
T (°C)	37.6	37.3	37.3	37.3	37.8	38.2	36.6	37	36.5	36.8	36.6	36.2	36.9	36.8	36.5
WBC (×10^9^/L)	19.8	14.1	9.96	8.17	14.46	18.01	18.02	7.4	4.78	8.44	3.65	3.11	8.15	4.6	2.96
PCT (ng/ml)	49.9	21.79		2.75	3.32		2.33	0.79	0.57	0.71	0.55			0.4	
Creatinine (μmol/L)	94.4	80.3	91.7	191.2	164	264.3	188.6	118.3	115.5	151.5	116.1		137.1		148.8
Fungal D-glucans (pg/ml)		660.49		53.94	-	-	-	89.31	141.28	198.9		64.83	72.02	-	
Rhizopus microsporum by NGS			49,235			274		0		0					
Antifungal drug	L-AmB	L-AmB	L-AmB	L-AmB	L-AmB	L-AmB	L-AmB	L-AmB	L-AmB	L-AmB	ISA	ISA	ISA	ISA	ISA
Dosage	10 mg/kg	10 mg/kg	10 mg/kg	10 mg/kg	10 mg/kg	10 mg/kg	5 mg/kg	5 mg/kg	5 mg/kg	5 mg/kg	200 mg q8h	200 mg qd	200 mg qd	200 mg qd	200 mg qd
Blood potassium (mmlo/L)		4.1	4	4.5	4	4.3	4.3	4		4.3	4.3	3.4	4	3.8	3.9

T, body temperature; WBC, white blood cells; PCT, procalcitonin; L-AmB, liposomal amphotericin B; ISA, isaconazole.

**Figure 1:**
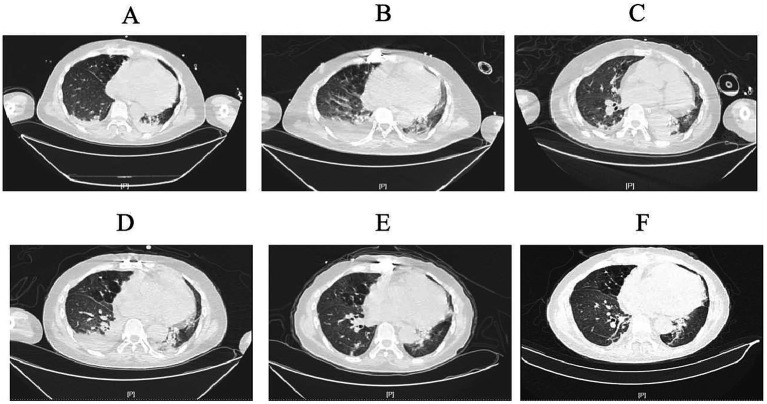
Computed tomography images. **(A)** (March 30th): Initial imaging showed scattered inflammatory infiltrates in both lungs, partial atelectasis of the lower lobes, and minimal bilateral pleural effusion. **(B)** (April 7th): Progressive inflammatory changes with scattered nodules in both lungs, slight worsening of inflammation, persistent pleural effusion, and increased right-sided effusion volume. **(C)** (April 17th): Stable inflammatory changes in both lungs, persistent lower lobe atelectasis, and reduced right-sided pleural effusion. **(D)** (April 22nd): Imaging consistent with prior findings, showing persistent inflammatory changes, pleural effusion, and partial atelectasis. Right-sided effusion volume increased. **(E)** (May 6th): Gradual resolution of inflammation, improvement in lower lobe atelectasis, and reduced pleural effusion volume. **(F)** (May 10th): Ongoing resolution of inflammatory changes, partial atelectasis, and minimal right-sided pleural effusion with slight volume increase compared to prior scan.

By March 29th, the patient’s exhibited worsening infection markers and a thrombus at the ECMO site in the right lower limb, leading to ECMO removal. L-AMB dosage was adjusted to 5 mg/kg daily after clinical pharmacy consultation. Chest CT revealed bilateral pulmonary inflammation, atelectasis, and minimal pleural effusion (Figure 1A).

On April 7th, the patient’s condition slightly deteriorated, with chest CT showing increased inflammatory changes (Figure 1B). Bronchoalveolar lavage fluid NGS results showed *Pseudomonas aeruginosa* and viral pathogens, prompting the addition of polymyxin B nebulization and ganciclovir for mucormycosis.

On April 17th, the patient’s expectorated sputum cultures showed CRPA. A chest CT showed scattered inflammatory changes in both lungs (Figure 1C). Despite treatment with polymyxin B, the sputum cultures still showed CRPA, and there was no significant improvement on the chest CT. Considering the poor efficacy of polymyxin B and the absence of *Rhizopus arrhizus* in the last two NGS results, polymyxin B (intravenous), amphotericin B cholesterol sulfate complex nebulization were discontinued. Instead, the patient was given ceftazidime-avibactam (2.5 g/8 h by intravenous drip), polymyxin B nebulization and oral isavuconazole.

By April 20th, the patient developed leukopenia, attributed to ganciclovir, which was subsequently discontinued. On April 22nd (Figure 1D), the patient’s condition improved with reduced *Pseudomonas aeruginosa* sequences on NGS, confirming the efficacy of the current regimen. Antimicrobial de-escalation was performed on May 6th (Figure 1E), transitioning to piperacillin-tazobactam (4.5 g/12 h) based on clinical improvement and infection marker trends. A follow-up chest CT on May 10th demonstrated further resolution of inflammation (Figure 1F), and the patient was discharged on oral isavuconazole.

## Discussion

Mucormycosis is associated with high mortality and requires early diagnosis and treatment (4, 5). Guidelines recommend surgical intervention and liposomal amphotericin B as first-line therapy. Studies have demonstrated that L-AMB are safe. In this case, a patient with pulmonary *Rhizopus arrhizus* infection post-cardiac transplantation was treated with high-dose liposomal amphotericin B (10 mg/kg/day) while on ECMO. Despite ECMO-related challenges, such as increased drug distribution volume, circuit adsorption, and reduced pulmonary tissue penetration, the regimen effectively controlled the infection (6–8), with *Rhizopus arrhizus* sequence counts decreasing to negative and no drug-related adverse reactions observed (9–12). The principal novelty of our report lies in its practical contribution to antifungal dosing during ECMO in a heart-transplant recipient who is already profoundly immunosuppressed. Traditional guidelines do not specify how L-AMB should be dosed when ECMO is added, yet ECMO alters drug pharmacokinetics through sequestration, increased volume of distribution, and augmented clearance. By presenting detailed serial drug-level and clinical-outcome data from a real-world case, we provide clinicians with an empirically derived dose-adjustment reference for L-AMB under these complex conditions. To our knowledge, this is the first documented account that quantitatively links ECMO-driven pharmacokinetic changes to L-AMB dose optimization specifically in a post-transplant setting.

This case underscores the importance of early recognition, rapid pathogen identification via next-generation sequencing, and dose optimization in ECMO-supported patients. However, current research on liposomal amphotericin B pharmacokinetics during ECMO is limited to case reports, and further studies are needed to establish precise dose prediction models.

## Conclusion

This case describes a cardiac transplant patient who developed pulmonary *Rhizopus arrhizus* infection during ECMO support and was effectively treated with high-dose liposomal amphotericin B. The successful management highlights the importance of early recognition and intervention in high-risk post-transplant patients on ECMO, providing valuable clinical experience for managing severe mucormycosis in similar cases.

## Data Availability

The raw data supporting the conclusions of this article will be made available by the authors, without undue reservation.
